# SARS-CoV-2 variants of concern surveillance including Omicron using RT-PCR–based genotyping offers comparable performance to whole genome sequencing

**DOI:** 10.3389/fcimb.2022.960065

**Published:** 2022-11-03

**Authors:** Simone Vanoni, Arnoldas Matulevicius, Besard Avdiu, Giada Scantamburlo, Camilla Ulekleiv, Pius M. Brzoska, Manohar R. Furtado, Jelena D. M. Feenstra, Alain Rico, Manoj Gandhi, Elisabetta Giacobazzi, Elisa Masi, Markus Paulmichl, Charity Nofziger

**Affiliations:** ^1^ PharmGenetix GmbH, Niederalm/Anif, Austria; ^2^ Thermo Fisher Scientific, South San Francisco, CA, United States; ^3^ Azienda sanitaria dell’Alto Adige, Laboratorio di microbiologia e virologia, Bolzano, Italy; ^4^ Department of Personalised Medicine, Privatklinik Maria Hilf GmbH, Klagenfurt, Austria

**Keywords:** SARS-CoV-2, COVID-19, variants of concern, omicron, delta, alpha, whole genome sequencing, mutation panel

## Abstract

Known SARS-CoV-2 variants of concern (VOCs) can be detected and differentiated using an RT-PCR–based genotyping approach, which offers quicker time to result, lower cost, higher flexibility, and use of the same laboratory instrumentation for detection of SARS-CoV-2 when compared with whole genome sequencing (WGS). In the current study, we demonstrate how we applied a genotyping approach for identification of all VOCs and that such technique can offer comparable performance to WGS for identification of known SARS-CoV-2 VOCs, including more recent strains, Omicron BA.1 and BA.2.

## Introduction

Since the beginning of the coronavirus infectious disease-19 (COVID-19) pandemic, several SARS-CoV-2 variants emerged and quickly spread worldwide, progressively replacing the previously circulating strains. Based on the increased risk they pose to public health, they have been designated either as variants of concern (VOCs) or variants under monitoring (VUMs) (Faria et al., 2021; Leung et al., 2021; O'Dowd, 2021; Tegally et al., 2021). Currently, the World Health Organization has reported five VOCs, namely, Alpha (B.1.1.7), Beta (B.1.351), Gamma (P.1), Delta (B.1.617.2), and Omicron (B.1.1.529), and several VUMs, including Eta (B.1.525) and Kappa (B.1.617.1) (https://www.who.int/en/activities/tracking-SARS-CoV-2-variants). Whole genome sequencing (WGS) is the conventional method for strain identification and surveillance, but it is resource-intensive, is not easily scalable for testing large numbers of samples, and, with a time to results of ≥3 days, can limit swift reactions by public healthcare systems for spread containment. On the other hand, VOCs/VUMs are characterized by a limited number of mutations of interest (MOIs) in the spike protein leading to higher infectivity, transmissibility, and/or vaccine resistance of SARS-CoV-2 (Barnes et al., 2020; Baum et al., 2020; Starr et al., 2020; Weisblum et al., 2020; Liu et al., 2021; Cherian et al., 2021; Greaney et al., 2021; Zhou et al., 2021). MOIs are shared among the different VOCs/VUMs and also appear in different combinations with other mutations, and it is therefore possible to differentiate between the known VOCs/VUMs just by examining these key residues in the SARS-CoV-2 genome. In the current study, we demonstrated that selecting appropriate panels of RT-PCR–based genotyping assays designed on the current circulating VOCs/VUMs can be equally precise for surveillance of VOCs/VUMs as WGS. This strategy can provide variant information in less than 24 h, which is a significant advantage over WGS, potentially allowing quicker adaptation of public health countermeasures for spread containment.

## Results

During the first waves of the pandemic, we performed SARS-CoV-2 variant determination through RT-PCR–based genotyping assays and WGS on 1,095 de-identified samples collected between November 2020 and June 2021 in the Alpine region of Austria and Italy (Carinthia, Salzburg, and South Tyrol). Given the central role of winter tourism of the area, several VOCs have sequentially emerged during this period, including Alpha, Beta, Gamma, and Delta, with Tyrol province reporting one of the largest clusters of Beta variant (B.1.351) in Europe from January to March 2021 (Özkan E et al., 2021; Paetzold et al., 2022). The simultaneous circulation of several VOCs in this region highlighted the need to identify and differentiate VOCs in patient samples. The Alpha variant emerged during the winter period and caused a rapid rise in infection incidence in Austria and Italy, whereas the Delta variant emerged in early summer and led to a slower rising wave of infections (Amman et al., 2022). The samples were selected based on positive SARS-CoV-2 status as determined by routine diagnostic testing using the TaqPath™ COVID-19 CE-IVD RT-PCR kit and covered several epidemiological parameters such as age, sex, and multiple viral loads, up to the Ct value of ≤34 for the N protein target.

**Figure 1:**
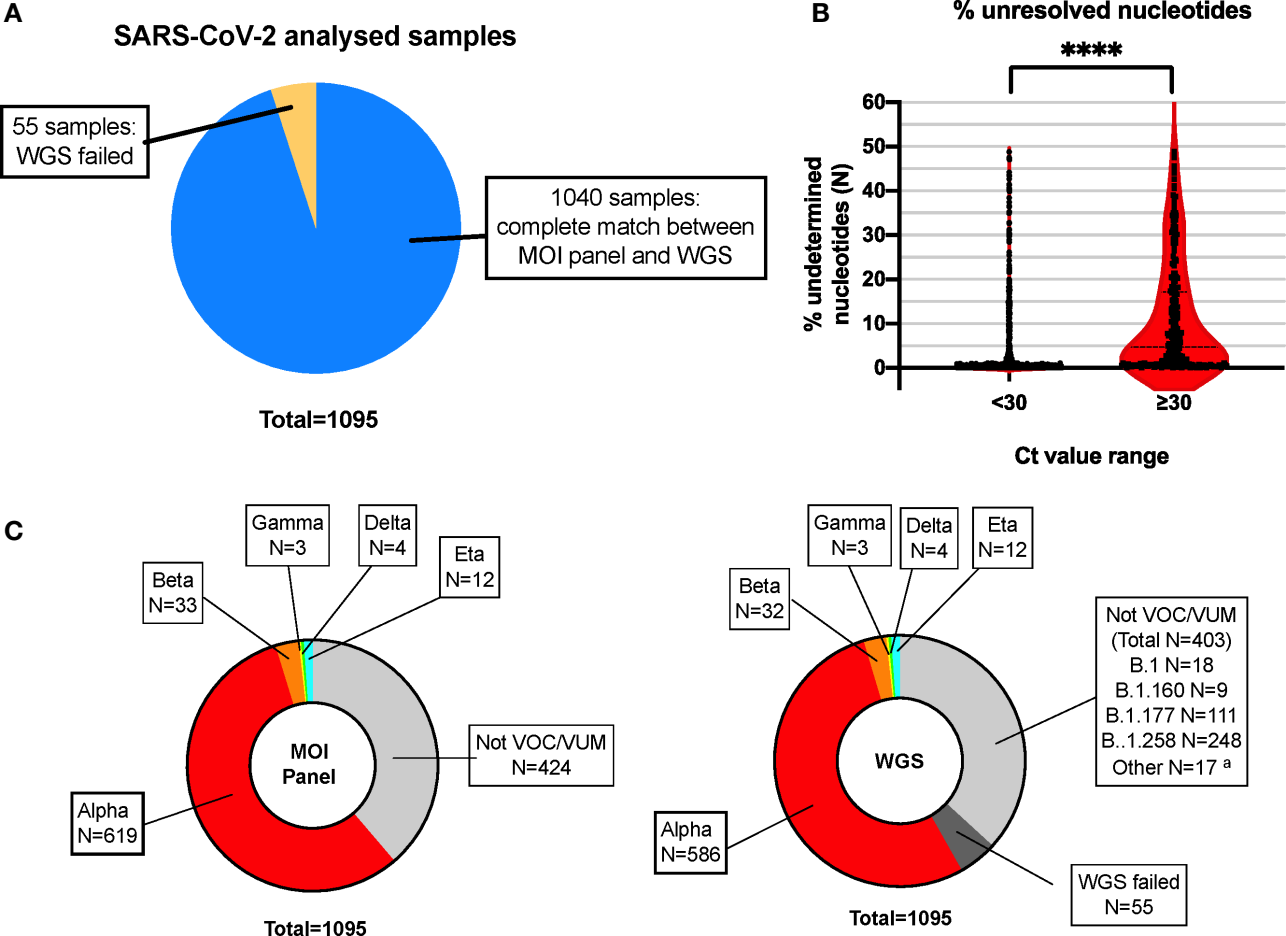
High concordance of RT-PCR–based MOI genotyping assays with WGS for SARS-CoV-2 variant detection. **(A)** Pie chart showing high concordance within the total number of samples analyzed in parallel with the MOI panel and WGS. N = 1,040 samples showed complete match in lineage identification between the two systems, while N = 55 did not pass QC for WGS and could not be unequivocally determined with such method. Blue: MOI panel/WGS match; orange: WGS failed. **(B)** Violin plot representing the percentage of unidentified bases (N-called nucleotides) due to low coverage areas following WGS, in samples with high (N-gene Ct < 30) or low (N-gene Ct ≥ 30) viral loads. Each plot represents a total of N = 779 and N = 261 for samples with Ct < 30 and Ct ≥ 30, respectively. ****p < 0.0001, as defined by unpaired *t*-test. **(C)** The MOI panel was able to detect and rule out all VOCs/VUMs, matching the WGS results, except for the 55 samples that did not pass QC for WGS and could not be determined (WGS failed). The indication of the lineage for these samples is indicated in the text box for reference. MOI, mutation of interest; WGS, whole genome sequencing; QC, quality control; VOC, variant of concern; VUM, variant under monitoring. ^a^Minor lineages identified: B.1.1 (5 samples), B.1.153 (3 samples), B.1.221 (3 samples), B.1.1.232 (1 sample), B.1.1.234 (1 sample), B.1.1.374 (1 sample), B.1.1.39 (1 sample), B.1.1.70 (1 sample), and C.36 (1 sample).

Based on the VOCs/VUMs circulating, an initial panel composed of 10 MOIs and the S gene target failure (SGTF) was chosen (**Table 1**). Furthermore, SGTF of the TaqPath™ COVID-19 CE-IVD RT PCR kit can be used as a proxy for the presence of S:delH69_V70. The lineage was successfully assigned in 671 out of 1,095 samples, and according to their MOI/SGTF fingerprint, the samples were Alpha (B.1.1.7, N = 619), Beta (B.1.351, N = 33), Gamma (P.1; N = 3), Delta (B.1.617.2, N = 4), and Eta (B.1.525, N = 12) variants (**Figure 1C**). The remaining 424 samples were determined not to represent VOCs/VUMs based on the MOI/SGTF profile.

**Table 1:** VOC/VUM determination using a panel of 10 genotyping assays for SARS-CoV-2 spike protein MOI and SGTF.

Panel Version 1		Spike protein mutations										
		del H69_V70	del L242_244	K417N	K417T	L452R	E484K	E484Q	N501Y	P681H	P681R	F888L
**VOC**	**Alpha** **B.1.1.7**	**mut**	**wt**	**wt**	**wt**	**wt**	**wt**	**wt**	**mut**	**mut**	** **	**wt**
	**Beta** **B.1.351**	**wt**	**mut**	**mut**	** **	**wt**	**mut**	** **	**mut**	**wt**	**wt**	**wt**
	**Gamma** **P.1**	**wt**	**wt**	** **	**mut**	**wt**	**mut**	** **	**mut**	**wt**	**wt**	**wt**
	**Delta** **B.1.617.2**	**wt**	**wt**	**wt**	**wt**	**mut**	**wt**	**wt**	**wt**	** **	**mut**	**wt**
**VUM**	**Eta** **B.1.525**	**mut**	**wt**	**wt**	**vwt**	**wt**	**mut**	** **	**wt**	**wt**	**wt**	**mut**
	**Kappa** **B.1.617.1**	**wt**	**wt**	**wt**	**wt**	**mut**	** **	**mut**	**wt**	** **	**mut**	**wt**

In order to provide visual aid, wild type (wt) fields are highlighted with a gray shade, while mutant (mut) fields are highlighted with a red shade.

**Figure 2:**
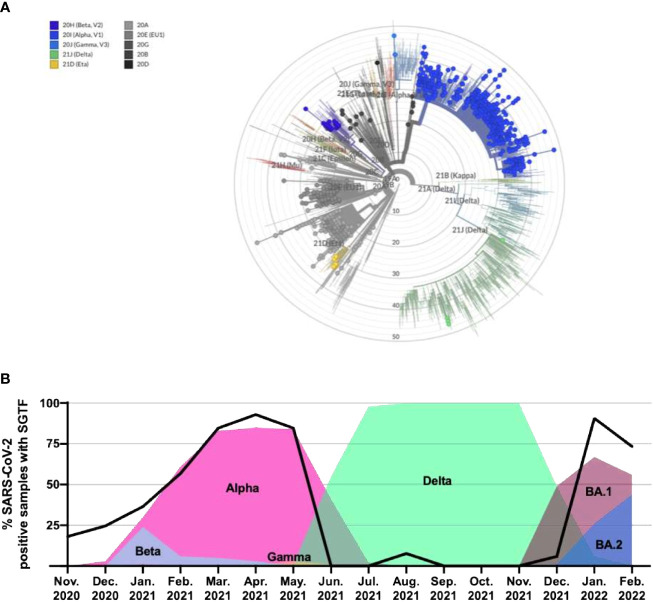
Phylogenetic tree of all identified lineages and SGTF in SARS-CoV-2–positive samples as a marker of alternating dominant VOCs with and without del69_70 in the S gene. **(A)** Nextclade phylogenetic radial tree from the Nextstrain global reference tree. Each of the circle represents 1 of the 1,040 SARS-CoV-2 genomes that passed quality control for WGS, whereas branch length represents nucleotide divergence. **(B)** Percentage of positive samples from November 2020 through February 2022 exhibiting the SGTF using the TaqPath™ COVID-19 CE-IVD RT-PCR kit and their linkage with novel VOC/Variant of Interest (VOI) appearance and spreading. SGTF, S gene target failure; VOC, variant of concern; WGS, whole genome sequencing.

Based on the WGS analysis, 637 samples were identified as VOCs/VUMs, whereas 403 samples belonged to 13 lineages not considered to pose a major risk to global public health (**Figure 1C**). The distribution of these 1,040 samples is shown as phylogenic tree of Nextstrain clades in **Figure 2A**. For the 637 VOC/VUM samples, the WGS results confirmed in 100% of the cases the lineages defined using the RT-PCR–based approach. Fifty-five samples, all with low viral load (N-gene Ct ≥ 30) and corresponding to the 5% of the total, did not pass quality control for WGS, and therefore, the SARS-CoV-2 lineages could not be determined, making a comparison with the genotyping panel not possible (**Figure 1A**). Furthermore, an analysis for unresolved nucleotides due to low sequencing coverage of the 1,040 samples that passed QC showed that the 261 analyzed samples with low viral load (N-gene Ct ≥ 30) had a significantly higher percentage of sequence gaps with respect to samples with higher viral loads (N-gene Ct < 30) (**Figure 1B**). Overall, these data demonstrate higher robustness of the MOI panel, in particular at low viral loads, when compared with WGS.

Following a period of dominance of the Delta SARS-CoV-2 VOC, in November 2021, the Omicron variant emerged. To assess the use of the genotyping approach for detection and differentiation of Omicron and Delta VOCs, we designed a new panel containing eight mutation assays (**Table 2**). One hundred two SARS-CoV-2–positive samples collected in Austria in the period of September 2021 to January 2022 were analyzed with the TaqMan assays and the lineages assigned based on the mutation profile and the SGTF status. When compared with WGS, the genotyping approach accurately assigned 100% of Delta and Omicron cases, with 55.8% (n = 57) identified as Delta, 41.2% (n = 42) as Omicron *BA.1-like*, and 3% (n = 3) as Omicron *BA.2-like*. Interestingly, WGS data showed that among the 58 Delta VOC samples, 15 different Pango sublineages could be detected, including AY.4.9, AY.43, and AY.46. This indicates that the genotyping approach can correctly assign Delta VOC on a wide range of sublineages. Similarly, for Omicron BA.1 and BA.2 strains, every sample was correctly identified regardless of the subvariant.

**Table 2:** VOC/VUM determination using an updated panel of eight genotyping assays for SARS-CoV-2 spike protein MOI and SGTF.

Panel Version 2		Spike protein mutations									
			del H69_V70	G339D	K417N	L452R	Q493R	N501Y	P681H	P681R	Q954H
**VOC**	**Omicron** **B.1.1.529**	**BA.1**	**mut**	**mut**	**mut**	**wt**	**mut**	**mut**	**mut**	**wt**	**mut**
		**BA.2**	**wt**	**mut**	**mut**	**wt**	**mut**	**mut**	**mut**	**wt**	**mut**
	**Delta** **B.1.617.2**		**wt**	**wt**	**wt**	**mut**	**wt**	**wt**	**wt**	**mut**	**wt**

White fields indicate that the presence of another mutation at the same position would cause the particular assay to show a result which can look as a heterozygous call and can therefore signal the presence of a different mutation at that position.In order to provide visual aid, wild type (wt) fields are highlighted with a gray shade, while mutant (mut) fields are highlighted with a red shade.

Of the 1,142 samples analyzed through WGS during this study, 923 displayed SGTF on the TaqPath™ COVID-19 CE-IVD RT-PCR kit. Accordingly, WGS confirmed the presence of S:delH69_V70 in all these samples, highlighting the fact that SGTF can be used as a reliable proxy for this deletion. In addition, we analyzed the SGTF occurrence in 12,000 SARS-CoV-2–positive samples as part of routine testing through the period of November 2020 until February 2022 (**Figure 2B**). Of these 12,000 samples, 4,312 (35.9%) exhibited SGTF. The percentage increased from 18.1% in November 2020 to 92.9% in April 2021, marking the appearance and later dominance of the Alpha (B.1.1.7) VOC and, to a lesser extent, the B.1.258 variant in the region, both of which contain S:delH69_V70. During the second half of 2021, between June and December, most samples did not exhibit SGTF when the Delta variant was dominant. SGTF reappeared with Omicron BA.1 in late December 2021 and in January 2022 (90.7%), with a slight decrease again during February (73.3%), compatible with the increasing presence of the BA.2 variant. Overall, this is indicating that the SGTF is a good proxy for VOC surveillance once they are identified and characterized.

## Discussion

Our study demonstrates the efficacy of an RT-PCR–based approach using a selected set of genotyping assays as an alternative to WGS for surveillance testing, with results obtained in <24 h, as opposed to WGS, which turnaround times range from 43 to 93 h (Plitnick et al., 2021), depending on the platform used, or down to 30 h with lower throughput (Fissel et al., 2022). The main difference resides in the fact that the genotyping approach avoids several steps needed for WGS (reverse transcription, library preparation, chip templating, and sequencing), with a gain of around 33 h only in instrument time and with similar throughput. Moreover, this approach requires only one instrument, which could be the same used for SARS-CoV-2 detection, thus allowing for quick VOC identification. This is especially important for those laboratories where a sequencer is not available. Because the panel can be modulated by adding, subtracting, or replacing individual MOI, it can be easily tailored to current relevant strains circulating in the region, including a built-in redundancy with at least two MOIs with each target strain. From an analytical standpoint, RT-PCR–based genotyping also allows samples with low viral loads (Ct ≥ 30) to be analyzed as compared with WGS which are generally limited to samples with higher viral loads (Ct < 30). This is especially important, because lower viral loads may be observed due to increasing vaccination rates (Levine-Tiefenbrun et al., 2021; Thompson et al., 2021). Furthermore, as previously demonstrated for Alpha VOC (Volz et al., 2021) and most recently for Omicron VOC (Scott et al., 2021), the presence of the S-gene target failure in the SARS-CoV-2 detection assay could be a useful tool for the indirect surveillance of variants carrying the S:delH69_V70 mutation. However, a limitation of the RT-PCR–based approach remains—that it can only be applied to detect known VOCs or MOIs and cannot replace, but rather complement WGS in that context. Similar to the seasonal flu, surveillance testing for SARS-CoV-2 is going to be critical as COVID-19 evolves from a pandemic to an endemic state. Rapid and large-scale strain information availability, especially for those lineages triggering breakthrough infections and capable of causing disease in convalescent and/or vaccinated populations, will be important for public health institutions in decisions around vaccine modifications, the possibility of recurring to monoclonal antibody treatments for hospitalization prevention, and the containment measures to limit spread of such variants in the population. RT-PCR–based mutation panels allow quicker surveillance testing on a significantly larger number of samples and could be seen as a complementary approach to WGS, allowing laboratories to reserve precious sequencing resources for novel variant identification.

## Methods

### Sample collection and processing

Naso- or oropharyngeal samples representing leftover clinical specimens collected and delivered as swabs stored in viral transport media (VTM) from November 2020 until February 2022 from three different regions of Austria and Italy (Salzburg, Carinthia, and South Tyrol) were included in the study. All samples were collected and analyzed under the direction and/or the supervision of the respective public healthcare systems. RNA was extracted using the QIAmp viral RNA Mini kit (QIAGEN GmbH), a silica membrane system for viral RNA isolation, according to the manufacturer´s instructions. Briefly, 140 µl of the specimen VTM were transferred into 560-µl lysis buffer containing carrier RNA and MS2 Phage as an internal control for RNA extraction and then incubated for 10 min at room temperature for viral inactivation and lysis. Ethanol (560 µl) was then added before column loading, allowing RNA binding to the silica membrane. Following two sequential washes of the membrane, high-quality RNA was then eluted in RNAse-free buffer and immediately used for downstream analyses.

### SARS-CoV-2 RT-PCR–based variant detection analysis

Two customized mutation panels consisting respectively of 10 and 8 MOI assays in the S-gene were tested on the QuantStudio 12K Flex PCR system using the Design & Analysis software v.2.5.1 (Thermo Fisher Scientific). The first panel was composed of the following MOIs detected by assays from the TaqMan™ SARS-CoV-2 mutation panel (Thermo Fisher Scientific, catalogue numbers of each assay listed in brackets): L242_244Del (CV2W7KX), K417N (CV2W7KX), K417T (A51815), L452R (A51819), E484K (A51813), E484Q (A51820), N501Y (A51812), P681H (A51816), P681R (A51822), and F888L (A51827). The second version of the panel contained the following mutations and corresponding assays: G339D (CV47VRX), K417N (CV2W7KX), L452R (A51819), Q493R (CV47VRX), N501Y(A51812), P681H (A51816), P681R (A51822), and Q954H (CVAAAAH). The assays were used with the TaqPath™ 1-Step RT-qPCR Master Mix, CG (Thermo Fisher Scientific, Waltham, MA, USA; Cat. No. A15300) and the RNA extracted for detection of SARS-CoV-2. The presence or absence of S:delH69_V70 was determined based on SGTF using the TaqPath™ COVID-19 CE-IVD RT-PCR kit on the QuantStudio 12K Flex PCR system and the Expression Suite software version 1.3 (Thermo Fisher Scientific).

### SARS-CoV-2 whole viral genome sequencing

Viral RNA was reverse-transcribed using the Invitrogen™ SuperScript™ VILO™ cDNA Synthesis kit (Thermo Fisher Scientific). All 1,197 samples included in the study were subjected to WGS library preparation using the Ion AmpliSeq™ Library Kit Plus kit, and sequencing was performed on the Ion GeneStudio™ S5 System running Ion 540™ Chips (Thermo Fisher Scientific) for 550 flows. For details on library preparation and sequencing run, see SI Appendix.

### NGS data analysis, metrics, and lineage identification

Reads were aligned with the Ion AmpliSeq SARS-CoV-2 reference sequence based on the Wuhan-Hu-1 NCBI reference genome (accession number: MN908947.3) using the pipeline on the Torrent Suite (v.5.12.2) and the following plugins, all with their default parameters: SARS_CoV_2 variantCaller (v.5.16.0.5) with the “Generic-S5/S5XL (540)-Germ Line-Low Stringency” for identification and analysis of mutations, SARS_CoV_2_coverageAnalysis (v.5.16.0.4) for the specific SARS-CoV-2 sequence coverage analysis, SARS_CoV_2_annotateSnpEff (v.5.16.0.5) for the annotation of the mutations identified on each sequence and prediction of effects, and IRMAreport (v.1.3.0.2) and generateConsensus (v.5.16.0.10) for the creation of the consensus sequences in FASTA format. Data passing quality filters were applied prior lineage assignment. Only those samples with ≥10,000 mapped reads, a mean depth of ≥30, and a number of unresolved nucleotides (N-called bases) <50% of the sequence as defined by the generateConsensus plugin were chosen for lineage analysis. One thousand forty samples passed the quality filters, with a median (IQR) of 587,586 (694,159) mapped reads, 2,035 (4,396) mean depth, and 90.5% (11.4%) of uniformity. Unambiguous Pango lineage identification of all the 1,040 samples passing controls was obtained using the SARS-CoV-2-lineageID plugin (v.5.16.0.5-20210519) and the Pangolin COVID-19 Lineage Assigner (https://pangolin.cog-uk.io), software version 2.4.2, starting from the generateConsensus output. All the consensus sequences for each analyzed sample were uploaded and are available on GISAID, a public-domain archive collecting and sharing data from all influenza viruses and coronavirus causing COVID-19 (Khare et al., 2021). All the accession ID numbers are reported in **Table S1**.

## Data availability statement

The datasets presented in this study can be found in online repositories. The names of the repository/repositories and accession number(s) can be found in the article/**Supplementary Material**.

## Ethics statement

Ethical review and approval was not required for the study of human participants in accordance with the local legislation and institutional requirements. Written informed consent from the patients/participants was not required to participate in this study in accordance with the national legislation and the institutional requirements.

## Author contributions

SV, CU, JF, MG, MP, and CN participated in the study conceptualization and experimental design. SV, JF, MG, MP, and CN drafted the manuscript. SV, AM, BA, and GS performed the experiments. SV, CU, PB, MF, JF, AR, MG, MP, and CN participated in data analysis and/or interpretation. EG and EM contributed in samples collection. All authors contributed to the article and approved the submitted version.

## Funding

Pharmgenetix GmbH acknowledges the Österreichische Forschungsförderungsgesellschaft GmbH (FFG) for support via the PGx-Next-Generation Analytics Part 2 grant (FO0999891633/42175800).

## Conflict of interest

SV, AM, BA, GS, and CN are employees of PharmGenetix GmbH. CU, PB, MF, JF, AR, and MG are employees of Thermo Fisher Scientific. MP is employed by Privatklinik Maria Hilf GmbH.

The remaining authors declare that the research was conducted in the absence of any commercial or financial relationships that could be construed as a potential conflict of interest. Thermo Fisher Scientific provided the reagents for the study.

## Publisher’s note

All claims expressed in this article are solely those of the authors and do not necessarily represent those of their affiliated organizations, or those of the publisher, the editors and the reviewers. Any product that may be evaluated in this article, or claim that may be made by its manufacturer, is not guaranteed or endorsed by the publisher.
