# Supplementary material for: SARS-CoV-2 variants of concern surveillance including Omicron using RT-PCR–based genotyping offers comparable performance to whole genome sequencing
Source: Front Cell Infect Microbiol. 2022 Nov 3;12:960065. doi: 10.3389/fcimb.2022.960065 (PMC9670535; doi:10.3389/fcimb.2022.960065)
Supplement: Supplementary file 1 [file DataSheet_1.docx]

Supplementary Material - Rapid detection of SARS-CoV-2 variants of concern using a RT-PCR based mutation panel

# Supplementary Data

**Extended Methods**

**SARS-CoV-2 RT-PCR-based variant detection analysis**

Two customized TaqMan™ SARS-CoV-2 mutation panels (Thermo Fisher Scientific, Waltham, CA, USA) were used to determine the SARS-CoV-2 variant status in 1197 samples included in the study. Each assay of the panel consists of specific primers for amplifying the target sequence region and two labeled probes to detect either the reference (wild-type) (VIC™ dye) or the mutant (FAM™ dye) sequence. The first panel targeted 10 MOIs within the S gene: L242_244Del, K417N, K417T, L452R, E484K, E484Q, N501Y, P681H, P681R and F888L, and was used to target the VOCs present during the first waves (Alpha, Beta, Gamma, Delta and Eta). The second panel contained 8 MOIs: G339D, K417N, L452R, Q493R, N501Y, P681H, P681R and Q954H, used to distinguish between Delta and Omicron variants. All analyses were carried out using the QuantStudio 12K Flex PCR system (Thermo Fisher Scientific) according to the following protocol: briefly, 5µL isolated viral RNA were combined in 384-well plates with 2.5µL 4x TaqPath™ 1-Step RT-qPCR Master Mix CG, 0.25µL 40x TaqMan SARS-CoV-2 Mutation Panel Assay and 2.25µL nuclease-free water. RT-PCR amplification was performed with the following parameters: 10 minutes at 50°C, 2 minutes at 95°C, followed by 45 cycles of 95°C for 3 seconds and 60°C for 30 seconds. A 30 second post-read detection at 60°C was set in order to detect the end-point signal for each assay. All the data obtained were analyzed using the Design & Analysis Software version 2.5.1 (Thermo Fisher Scientific), and genotype was assessed using the allelic discrimination plot: samples clustering along the X-axis were identified as carrying the reference (wild-type) allele (Allele 1, VIC™ dye), while samples along the Y-axis were defined as mutant (Allele 2, FAM™ dye).

In addition to the 10 MOIs, the presence or absence of S:delH69_V70 was determined using the TaqPath™ COVID-19 CE-IVD RT-PCR Kit (Thermo Fisher Scientific), a multiplex RT-PCR assay targeting N, S and ORF1ab genes in the SARS-CoV-2 genome and normally used for routine diagnostic test. Presence of the S:delH69_V70A deletion is revealed by S gene target failure (SGTF), and was therefore used as a proxy for detection of this deletion in the samples (1, 2). The TaqPath™ COVID-19 CE-IVD RT PCR Kit (Thermo Fisher Scientific) was used according to manufacturer’s instructions except that the total reaction volume was reduced from 25µl to 12.5µl. All experiments were carried out on the QuantStudio 12K Flex PCR system (Thermo Fisher Scientific) and analyzed using the Expression Suite Software version 1.3 (Thermo Fisher Scientific). Samples were considered positive when at least two out of three target genes had Ct values <37.

**Complementary DNA (cDNA) synthesis**

Viral RNA from isolates was reverse transcribed (RT) using the Invitrogen™ SuperScript™ VILO™ cDNA Synthesis Kit (Thermo Fisher Scientific) according to the manual library preparation protocol on the Ion AmpliSeq™ SARS‐CoV‐2 Research Panel on an Ion GeneStudio™ S5 Series System Quick Reference (Thermo Fisher Scientific, MAN0019277, Rev. B.0). Briefly, 7µL of isolated and quantified viral RNA were combined with 2µL 5x VILO™ Reaction Mix and 1µL 10X SuperScript™ Enzyme mix. The RT reaction was performed on a Bioer 96-Well Thermal Cycler (Hangzhou Bioer Technology Co., Hangzhou, China) at 42°C for 30 minutes followed by 5 minutes at 85°C.

**Library preparation for SARS-CoV-2 Next Generation Sequencing (NGS)**

Whole viral genome sequencing was performed using the Ion AmpliSeq™ Library Kit Plus (Thermo Fisher Scientific) following the manufacturer´s instructions (Thermo Fisher Scientific, MAN0017003, Rev. C.0) with panel-specific modifications in accordance with the Ion AmpliSeq™ SARS‐CoV‐2 Research Panel on an Ion GeneStudio™ S5 Series System Quick Reference (Thermo Fisher Scientific, MAN0019277, Rev. B.0). In brief, sample specific master mixes were obtained by combining 10µL synthesized cDNA with 4.5µL 5X Ion AmpliSeq™ HiFi Mix and 3.5µL nuclease-free water. Each sample specific master mix was divided in two, and 2µL of each of the 5X Ion AmpliSeq™ Primer pool 1 and 2 were added to the corresponding half. Target amplification was performed with the following thermal cycling parameters: 2 minutes at 98°C, followed by 10-26 cycles at 98°C for 15 seconds and 60°C for 4 minutes. PCR cycles ranging from 10 to 26 were determined according to the calculated viral load as defined by the conversion table provided by the manufacturer, with the lowest cycles (10) for samples with ≥128000 viral copies/µL (N gene Ct value ≤17) and the highest cycles (26) for samples with 1 viral copy/µL (N gene Ct value = 34). Combined Primer pool 1 and 2 targeted amplification reactions were then partially digested and the Ion Xpress™ Barcode adapters ligated according to the manufacturer’s instructions. Following ligation, purification of the libraries was carried out by adding 45µL Agencourt™ AMPure™ XP Reagent (Beckman Coulter, Brea, CA, USA), washing twice with 70% Ethanol and then eluting in 50µL Low TE buffer. Diluted (1:100) Ion AmpliSeq™ Research Panel libraries were quantified using the Ion Library TaqMan® Quantitation Kit (Thermo Fisher Scientific) according to the manufacturer’s instructions. Library concentration was then calculated by multiplying 100-fold the concentration determined from the qPCR using the Design and Analysis software v.2.5.1 (Thermo Fisher Scientific) based on the standard curve dilutions. Barcoded libraries were diluted to 70pM, pooled in equal molar amounts and then loaded onto the Ion Chef™ instrument (Thermo Fisher Scientific) for emulsion PCR, enrichment and loading onto two Ion 540™ Chips (Thermo Fisher Scientific). Sequencing runs were all performed on the Ion GenStudio™ S5 System (Thermo Fisher Scientific), with each Chip ran for 550 flows.

**References**

1. Bal A, Destras G, Gaymard A, Stefic K, Marlet J, Eymieux S, et al. Two-step strategy for the identification of SARS-CoV-2 variant of concern 202012/01 and other variants with spike deletion H69-V70, France, August to December 2020. Euro Surveill. 2021;26(3).

2. Brown KA, Gubbay J, Hopkins J, Patel S, Buchan SA, Daneman N, et al. S-Gene Target Failure as a Marker of Variant B.1.1.7 Among SARS-CoV-2 Isolates in the Greater Toronto Area, December 2020 to March 2021. JAMA. 2021;325(20):2115-6.

**Figure legends**

**Table S1. Accession ID numbers of the SARS-CoV-2 RNA sequences uploaded on the GISAID database.** Accession ID, location, collection and submission date for each of the sequences uploaded on the GISAID database are reported in the table.
